# Psychometrics of the Functional Oral Intake Scale for Infants

**DOI:** 10.3389/fped.2019.00156

**Published:** 2019-04-18

**Authors:** You Gyoung Yi, Hyung-Ik Shin

**Affiliations:** ^1^Department of Rehabilitation Medicine, Veterans Medical Research Institute, Veterans Health Service Medical Center, Seoul, South Korea; ^2^Department of Rehabilitation Medicine, Seoul National University Hospital, Seoul National University College of Medicine, Seoul, South Korea

**Keywords:** eating abilities, infant, functional oral intake scale, videofluoroscopic swallowing study, oral feeding, nutrition

## Abstract

This study aimed to investigate the reliability and validity of the Functional Oral Intake Scale (FOIS) for infants. Infants (age, <1 year) who underwent a videofluoroscopic swallowing study (VFSS) were included in this retrospective study. Their nutrition records at the time of the VFSS were separately evaluated by two raters using the five-point FOIS for infants. Categorical swallowing and aspiration impairment scale data were also obtained from the VFSS. The inter-rater reliability of the FOIS for infants was high (95.5% absolute agreement) among the 201 evaluated infants, and this scale was significantly correlated with aspiration severity in the VFSS. We also investigated whether infants with partial oral feeding (POF) at the FOIS evaluation had achieved full oral feeding within 1 year of the evaluation and used this information to estimate whether the caloric contribution, as well as consistency of oral feeding, affected the feeding outcomes. This analysis included 33 infants who were receiving both oral and tube feeding (i.e., POF). Among them, 26 infants achieved full oral feeding (FOF) without tube feeding after 1 year. Their initial contribution from oral feeding was higher than that in infants who still maintained POF after 1 year (28.46 ± 22.79 vs. 6.00 ± 5.45%, *p* < 0.001). The five-point FOIS for infants, which reflected the expansion of their oral diet with growth, had adequate reliability and validity. The caloric contribution as well as consistency of oral feeding could be used to distinguish FOIS levels 2 and 3, which correspond to the POF status in infants.

## Introduction

Interventions for infants with dysphagia, such as environmental modifications (1), oral-motor stimulation (2), altered feeding routines (3), and neuromuscular electrical stimulation (4), have attracted increased attention both in clinical and research perspectives. Although such infants often require tube feeding to achieve a satisfactory caloric intake, this practice may lead to later feeding difficulties (5). Especially, in the case of starting tube feeding during the first year of life, it has been reported that the feeding outcome was poor although the pharyngeal phase of swallowing function is well preserved. Therefore, oral feeding in this era of tube feeding is recommended and encouraged (6). These earlier findings underscore the current paucity of and need for validated tools to measure the effects of these interventions and describe the swallowing status of infants.

The Functional Oral Intake Scale (FOIS, Table 1) was initially developed for the clinical documentation of changes in the functional oral intakes of liquids and foods by stroke patients (7). This seven-point observer rating scale is considered a reliable and valid tool that can be applied without placing an additional burden on the patient. In adults with dysphagia, the FOIS has been reported to correlate significantly with the Food Intake Level scale (8), swallowing item of the Functional Assessment Measure (9), Mann Assessment of Swallowing Ability, modified Barthel Index, modified Rankin scale, and dysphagia and aspiration during a videofluoroscopic swallowing study (VFSS) (7). Despite the wide use of the FOIS to evaluate dysphagia and assess oral intake recovery in adults (10), it has not been validated for use in infants.

**Table 1:** The functional oral intake scale according to Crary et al. (7).

Level 1	Nothing by mouth
Level 2	Tube-dependent with minimal attempts of food or liquids
Level 3	Tube-dependent with consistent oral intake of food or liquids
Level 4	Total oral diet of a single consistency
Level 5	Total oral diet with multiple consistencies but requiring special preparations or compensations
Level 6	Total oral diet with multiple consistencies without special preparation but with specific food limitations
Level 7	Total oral diet with no restrictions

The direct application of the FOIS to infants is challenging, as they are developing rapidly and will experience an expansion of the oral diet with age (11–13). Additionally, it can be difficult to distinguish FOIS level 2 (tube feeding with minimal attempts of oral feeding) from FOIS level 3 (tube feeding with consistent oral feeding) because consistent but very small amounts of oral feeding are possible during the period of tube feeding (14). Accordingly, Coppens et al. modified the FOIS (Table 2) for the evaluation of infants subjected to esophageal atresia repair by reducing the FOIS levels from seven to five stages to reflect the food expansion status (15). However, the authors did not report the validity or reliability of this modified scale. Therefore, the present study aimed to investigate the reliability and validity of this modified FOIS for infants. Additionally, we evaluated whether the oral feeding amount and frequency could be used to distinguish FOIS levels 2 and 3.

**Table 2:** The modified functional oral intake scale for infants according Coppens et al. (15).

	**Intake**
Level 1	Nothing by mouth
Level 2	Tube dependent with minimal attempts of food or liquids
Level 3	Tube dependent with consistent oral intake of food or liquids
Levels 4–6	Expansion of oral diet not reached^a^
Level 7	Expansion of oral diet reached^a^

a *Normal expansion of oral diet was considered reached when introduction of solid foods in pureed form started before 9 months of age and the introduction of mashed foods and soft lumps started before 12 months of age*.

## Materials and Methods

All study-related procedures were performed in accordance with the ethical standards of the institutional and/or national research committee and the 1964 Declaration of Helsinki. Ethical approval for the study was obtained from the Seoul National University Hospital Institutional Review Board (IRB) (No. 1807-189-963), which waived the requirement for informed consent due to the retrospective nature of the study. The following inclusion criteria were applied to potential subjects: (1) participation in the VFSS to evaluate a swallowing disorder at ≤1 year of age between 2011 and 2017 and (2) recording of the dietary status at the time of the VFSS by a nutritionist.

### FOIS for Infants

A seven-point ordinal FOIS has been validated in adults (Table 1) (7). As noted in the Introduction, a modified five-point version of the FOIS was developed to account for normal infant development (Table 2) (16). At FOIS levels 1, 2, and 3, the same criteria as those for adults were used in this study. However, we divided full oral feeding (FOF) into two categories: (1) achievement of oral diet expansion, the initiation of pureed foods before 9 months, and the initiation of mashed foods and those with soft lumps before 12 months as normal developmental stages; and (2) no achievement of this oral diet expansion.

### Inter-rater Reliability

The infants' caregivers were interviewed by a nutritionist, who recorded the type, amount, and consistency of food and liquid intakes, tube dependency, and total nutrient intake. Two occupational therapists with >2 years of experience in swallowing therapy retrospectively reviewed the nutritionist's medical records and assigned FOIS levels.

### Validity

Cross-validity was determined by comparing the infantile FOIS scores with the categorical ratings of swallowing impairment/aspiration severity and on the basis of the presence of swallowing impairment/aspiration determined by the VFSS (16). These tools were also used to validate the original FOIS (7). The swallowing impairment scale score was rated as 5 (normal) in the absence of a swallowing abnormality and as 1 (complete) if there was no response to a food stimulus. The aspiration impairment scale score was rated as 5 (normal) if the contrast material did not enter the true vocal cord and as 1 (complete) if the infant showed frank aspiration without reflex coughing (Table 3).

**Table 3:** Videofluoroscopic diagnostic criteria for dysphagia and aspiration, adopted from Mann et al. (16).

**SWALLOWING IMPAIRMENT (DYSPHAGIA)**	
Normal	No swallowing abnormality detected
Mild	Slight delay in bolus control, initiation of swallow, or transport, resulting in some stasis of material without laryngeal penetration
Moderate	Moderate delay in bolus control, initiation of swallow, or transport, resulting in coating or stasis of materials within the oral cavity and/or pharynx, slight laryngeal penetration, or trace aspiration of thin liquid only
Severe	Substantial delay in bolus control, initiation of swallow, and transport; significant (>10% of bolus) penetration and/or aspiration of one or all consistencies
Complete	No response to food stimulus; initiation of the swallow sequence is not obtained over several trials
**ASPIRATION**	
Normal	No entry of contrast material through the true vocal cords
Mild	Trace entry of contrast materials through the vocal cords
Moderate	Entry of <10% of the bolus through the true vocal cords
Severe	Entry of >10% of the bolus through the true vocal cords
Complete	Frank aspiration of materials through the vocal cords without an observable reaction by the patient

### Nutritional Contribution of Oral Feeding in Infants With Partial Oral Feeding

For infants with partial oral feeding (POF), the calorie contribution of oral feeding to the total caloric intake was estimated based on the same records used for the FOIS evaluation. Calories were calculated using the web version of CAN-Pro 5.0 software (http://www.kns.or.kr/English/index.asp, The Korean Science and Technology Center, Gangnam-gu, Seoul, Korea), which was developed by the Korean Nutrition Society for the nutritional evaluation of individuals or groups. If any food was not registered in the program, calories were calculated from the information printed on the product container. We also investigated whether infants with POF at the FOIS evaluation had achieved FOF within 1 year of the evaluation and used this information to estimate whether the caloric contribution, as well as consistency of oral feeding, affected the feeding outcomes.

### Statistics

For the five-point FOIS for infants, Cohen's κ and Cronbach's α coefficient were calculated as measures of the inter-rater reliability between the two evaluators. To assess cross-validity, Spearman's ρ-test was used to assess correlations between the FOIS for infants with swallowing impairments and aspiration severity ratings. We used Cramer's V (dichotomized data) to determine the association between the FOIS for infants and the presence or absence of swallowing impairment and aspiration.

Infants with POF were stratified according to whether they achieved FOF or not at 1 year after the evaluation, as determined by the caloric contribution of the oral intake at the time of the initial FOIS evaluation. Differences between these two groups were analyzed using an independent *t*-test. *P* < 0.05 was considered statistically significant. Analyses were performed using SPSS ver. 23.0 (IBM Corporation, Armonk, NY, USA).

## Results

### Subjects

Data were obtained from 201 infants (mean age: 199 days, range: 22–364 days) who underwent a VFSS between 2011 and 2017. The baseline characteristics and main diagnoses of the subjects are presented in Table 4. Brain lesions were found in 63 infants, which included hypoxic ischemic encephalopathy, encephalitis, intracerebral hemorrhage, brain tumor, corpus callosal dysgenesis, and hydrocephalus. Myopathy/motor neuron disease including spinal muscular atrophy, mitochondrial myopathy, myotubular myopathy, Fukuyama congenital muscular dystrophy, and congenital muscular dystrophy was found in 21 infants. The non-oral feeding, POF, and FOF groups did not differ significantly in age at the time of FOIS evaluation. VFSS findings with aspiration of liquid were found in 61 infants among 201 infants. The swallowing impairment scale (Table 3) scores ranged from 1 to 5 with a median of 4 (interquartile range: 3–4) with 38 infants having a score of 5, 78 with a score of 4, 53 with a score of 3, 31 with a score of 2, and 1 with a score of 1.

**Table 4:** Characteristics of subjects at the time of the videofluoroscopic swallowing study.

	**Eating abilities in infants**		
**Characteristics**	**Non-oral feeding** **(*n* = 80)**	**Partial oral feeding** **(*n* = 44)**	**Full oral feeding** **(*n* = 77)**
Female sex (%)	38 (47.5)	21 (47.7)	36 (46.8)
Age (range), days	171 (22–364)	233 (65–349)	210 (53–364)
**Main diagnosis**, ***n*** **(%)**			
Brain lesion	25 (31.3)	8 (18.2)	30 (39.0)
Myopathy/motor neuron disease	10 (12.5)	5 (11.4)	6 (7.8)
Gastrointestinal	6 (7.5)	6 (13.6)	7 (9.1)
Cardiac	4 (5.0)	3 (6.8)	5 (6.5)
Otolaryngology	7 (8.8)	4 (9.1)	10 (13.0)
Metabolic	6 (7.5)	0 (0)	1 (1.3)
Pulmonary	4 (5.0)	3 (6.8)	4 (5.2)
Immunologic	0 (0)	0 (0)	2 (2.6)
Unknown	1 (1.3)	0 (0)	1 (1.3)
Syndrome	17 (21.3)	15 (34.1)	11 (14.3)
Pierre Robin Syndrome	1	2	2
Kabuki syndrome	1	1	
Zellweger syndrome	1		1
Beckwith-Weidemann syndrome			1
Schinzel-Giedion syndrome			1
VACTERL syndrome	1		
Cornelia de lange syndrome		2	
Patau syndrome	1		
Sotos syndrome			3
Noonan syndrome	1		
Down syndrome		1	1
Miller-Dieker syndrome	1		1
Mobius syndrome		1	
CHARGE syndrome	2	2	
Treacher Collins syndrome	1	1	
Russel Silver syndrome	1		
Prader Willi syndrome	2		
Goldenhar syndrome	1	2	
CATCH 22 syndrome	3	1	
Smith-limli-opitz syndrome		1	
Wolf Hirschhorn syndrome			1
Mosaic 22q13 deletion syndrome		1	

### Inter-rater Reliability of the FOIS for Infants

The two occupational therapists achieved a high level of absolute agreement (95.5%) when applying the FOIS for infants (Table 2), as shown in Table 5 (κ = 0.935; intraclass correlation coefficient [ICC] = 0.996; 95% confidence interval [CI]: 0.995–0.997). The main disagreements were observed between FOIS levels 2 and 3 (*n* = 3) and levels 4 and 5 (*n* = 5).

**Table 5:** Inter-rater reliability of the FOIS for infants.

	**Rater 2**					
**Rater 1**	**1**	**2**	**3**	**4**	**5**	**Total**
1	79	1^a^	0	0	0	80
2	0	2	1	0	0	3
3	0	2	39	0	0	41
4	0	0	0	7	3	10
5	0	0	0	2	65	67
Total	79	5	40	9	68	201

*Shaded values indicate agreement between the evaluators*.

*FOIS, functional oral intake scale; TPN, total parenteral nutrition*.

a *TPN was performed without tube or oral feeding at the time of the examination. Rater 2 misinterpreted the meaning of TPN and classified it as FOIS level 2*.

### Validity

This study identified significant associations between the subject's level on the FOIS for infants and the presence (*p* = 0.014, *V* = 0.249) and severity (*p* = 0.001, *r* = 0.229) of aspiration during the VFSS. The infantile FOIS ratings correlated significantly with the severity (*p* = 0.040, *r* = 0.145), but not the presence of dysphagia (*p* = 0.188, *V* = 0.175).

### Nutritional Contribution of Oral Feeding in Infants With POF

**Figure 1:**
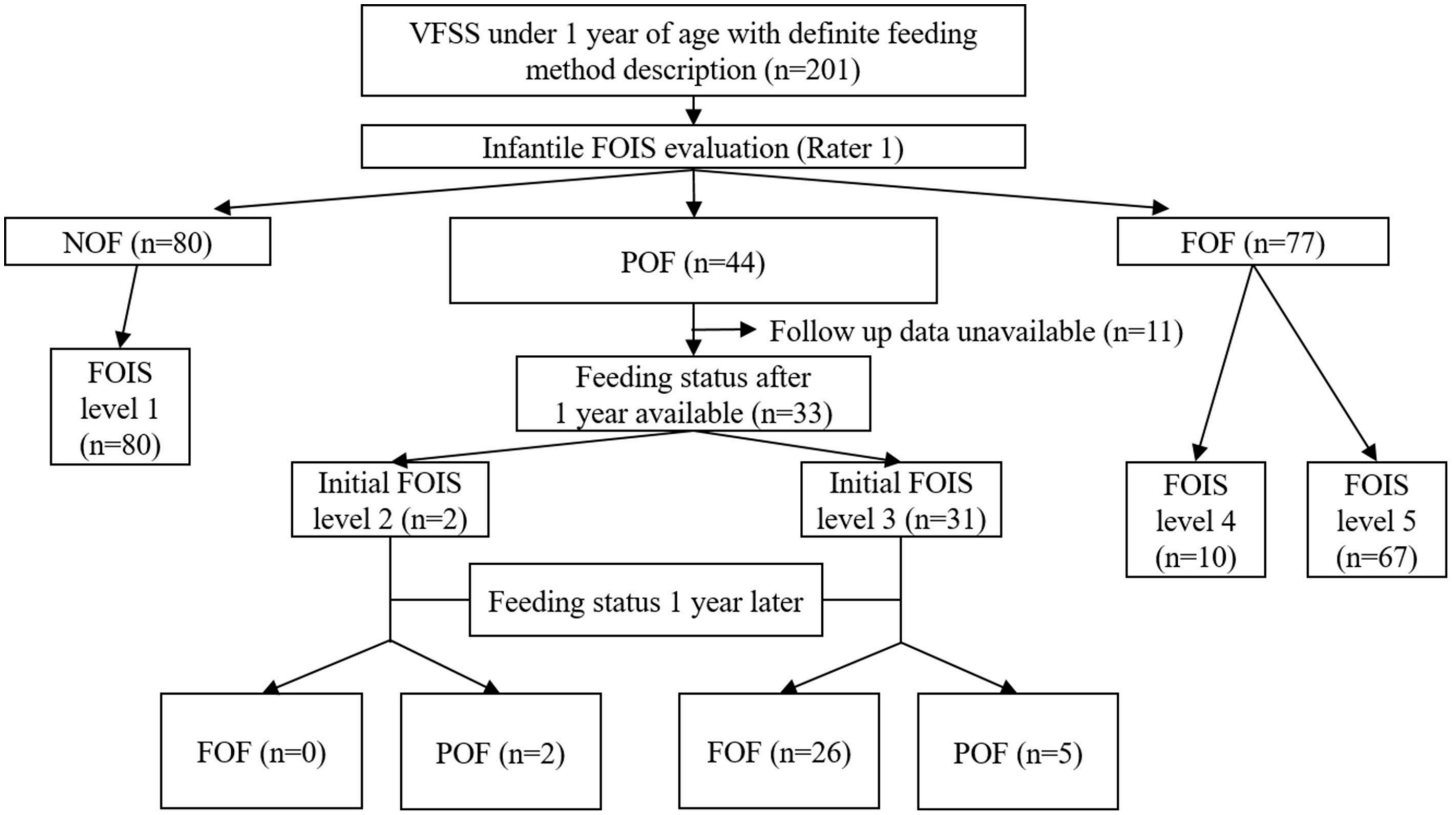
Acquisition of data relevant to the FOIS for infants and the feeding status after 1 year FOIS, functional oral intake scale; VFSS, videofluoroscopic swallowing study; NOF, non-oral feeding; POF, partial oral feeding; FOF, full oral feeding.

**Figure 2:**
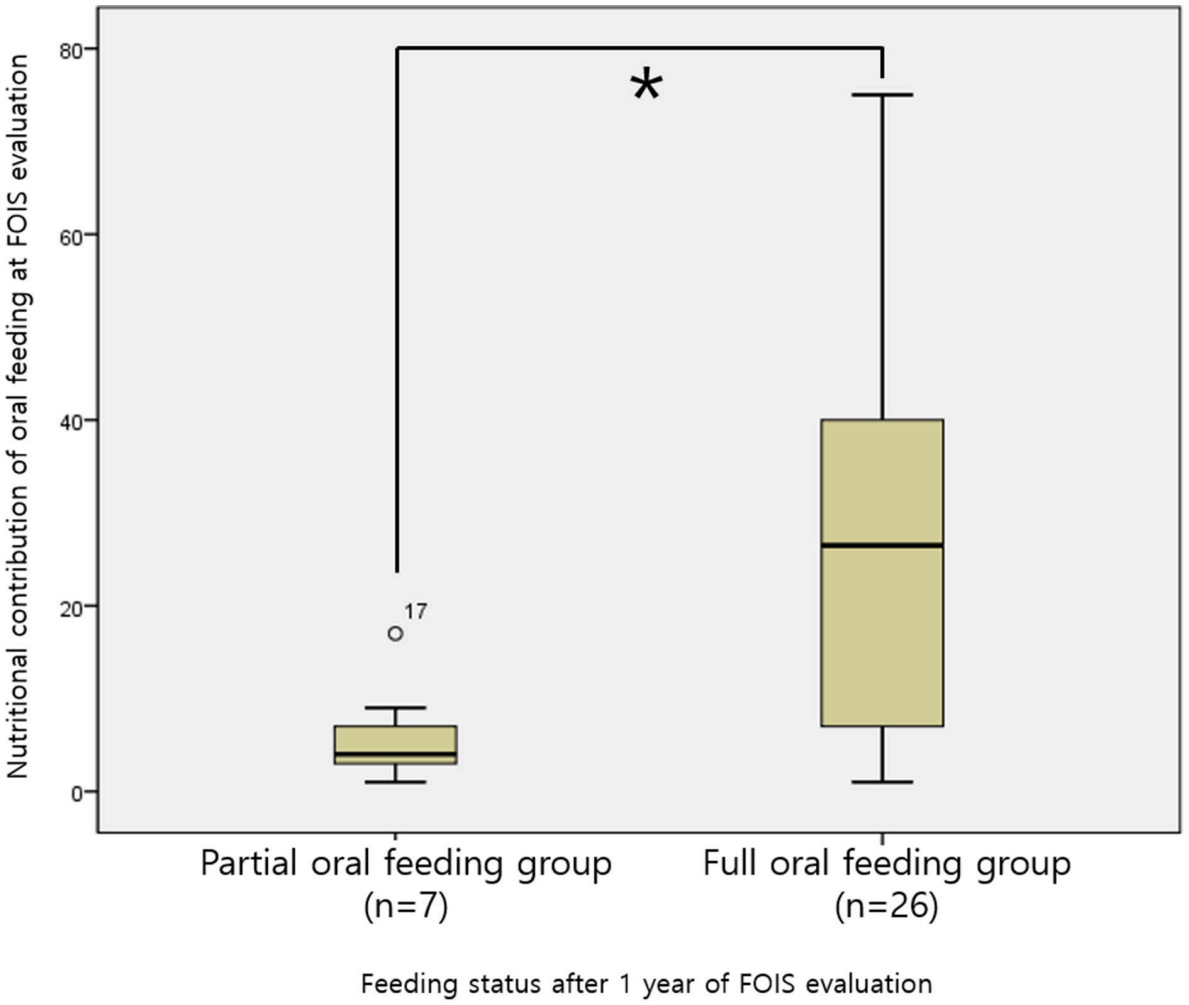
Comparison of the caloric contributions of oral intake among POF infants stratified according to the achievement or non-achievement of FOF after 1 year *p < 0.001. POF, partial oral feeding; FOF, full oral feeding.

This analysis included 33 infants who were receiving POF at the time of the VFSS and for whom nutritional records at 1 year after the VFSS were available (Figure 1). Among them, 26 infants achieved FOF after 1 year, and their mean nutritional contribution from oral feeding at the time of VFSS was 28.46 ± 22.79%, which was higher than 6.00 ± 5.45% in the seven infants who maintained a POF status (*p* < 0.001, Figure 2).

## Discussion

There is a need to aid clinicians in describing the feeding status and measuring outcomes for the management of infants with dysphagia. For example, therapeutic interventions such as oral sensorimotor stimulation for infants with oral hypersensitivity (17, 18) and fluid thickening to minimize aspiration symptoms (19, 20) necessitate describing the feeding outcome of the infants.

Accordingly, studies have validated the Neuromuscular Disease Swallowing Status Scale for children and adults (21, 22) and the Eating and Drinking Ability Classification System for children with cerebral palsy over a 3-year period (23, 24). However, in patient groups other than those described above, the adult version of the FOIS has been modified for the pediatric population.

Dodrill et al. (25) modified the FOIS to describe the swallowing function of infants/toddlers by replacing the levels indicating single/multiple consistency (level 4 and 5, respectively, in the adult version of the FOIS) with levels indicating requirement of modified liquids/solids (level 4 and 4.5, respectively, in the modified FOIS for infants). Strychowsky et al. utilized this version of the FOIS to describe the swallowing dysfunction among toddlers with laryngeal cleft (26). Christiaans et al. (4) also modified the original version of the FOIS by removing the level 4: total oral diet of a single consistency, in their report regarding the effectiveness of neuromuscular electrical stimulation in children with dysphagia. Later on, Baxter et al. applied the scale in children with esophageal atresia and tracheoesophageal fistulas (27).

However, these modified FOIS versions for the pediatric population have never been validated and tested for reliability. Additionally, these scales were proposed and used for young children as well as infants. Since oral diet expansions occur during the infantile period, the functional oral intake of infants should be assessed separately from that of young children. Therefore, we selected the modified FOIS that is specific to infants, proposed by Coppens et al. (15), and verified the validity and reliability of the scale. To our knowledge, this is the first study to validate the scale that was modified to measure food or liquid consumption by infants.

### Inter-rater Reliability and Validity

In this study, we observed a high level of inter-rater reliability for the FOIS for infants, which was similar to that in other studies. Among adult stroke patients, Crary et al. reported a high inter-rater reliability of the FOIS (absolute agreement, 85%) (7), and McMicken et al. reported ICC values of 0.975 and 0.964 at the time of admission and discharge, respectively (9). In the present study, the FOIS for children was associated with aspiration and dysphagia severity identified from the VFSS. This was similar to the results of a previous study, which evaluated the FOIS in stroke patients (7). Accordingly, the FOIS for infants may be appropriate for documenting feeding abilities and evaluating the effectiveness of interventions.

### Implication of the Distinction Between FOIS Levels 2 and 3 in Infants

Both FOIS levels 2 and 3 could be categorized as concurrent tube and oral feeding, and our observers reported three disagreements between these levels when evaluating patients in our study (Table 5). One patient was an 11-month-old infant with myotonic dystrophy who received a total tube feeding volume of 700 cc per day in five or six doses, as well as 20–40 g of puree once per day. One evaluator regarded once-daily feeding as a consistent oral intake (i.e., FOIS level 3), whereas the other considered it a minimal attempt at oral intake (i.e., FOIS level 2). The second patient was a 9-month-old infant with CHARGE syndrome who received a total tube feeding volume of 700 cc per day in four or five doses and attempted to consume minimal amounts of puree orally with every meal. The last patient was a 7-month-old infant with Pierre–Robin syndrome who received a total tube feeding volume of 800 cc per day in six or seven doses, together with a soft blended oral diet (~40 cc per day) at least once per day.

In our study, infants with POF who received a higher nutritional contribution from oral feeding were more likely to achieve FOF. This suggests that both the oral feeding amount and consistency should be considered when distinguishing FOIS levels 2 and 3. For example, eight out of 14 infants with <10% POF achieved FOF after 1 year, whereas 18 out of 19 infants with ≥10% POF achieved FOF after 1 year. Based on these results, we have revised the criteria for distinguishing FOIS levels 2 and 3 to consider both the oral intake amount and consistency, as shown in Table 6.

**Table 6:** The functional oral intake scale for infants considering both attempts and amounts of oral intake at level 2.

	**Intake**
Level 1	Nothing by mouth
Level 2	Tube-dependent with minimal oral intake^a^
Level 3	Tube and oral feeding in parallel^b^
Level 4	Expansion of oral diet not reached^c^
Level 5	Expansion of oral diet reached^c^

a *“Minimal oral intake” indicates minimal attempts of or a very small amount of oral intake*.

b *“In parallel” indicates consistent oral intake with significant caloric contribution*.

c *Normal expansion of oral diet is defined as the introduction of solid foods in pureed form before 9 months of age and the introduction of mashed foods and soft lumps before 12 months of age*.

### Implication of the Distinction Between FOIS Levels 4 and 5 in Infants

According to Pridham et al. infants can begin to consume semisolid food from a spoon between 5 and 7 months of age and complete this type of consumption at approximately 8 months of age (28). A 2001 guideline from the World Health Organization recommended the initiation of complementary feeding at 6 months of age and a concurrent and gradual solidification of foods (11). According to this guideline, the consumption of pureed, mashed, and semi-solid foods generally begins at 6 months of age, followed by the consumption of finger foods at 8 months and an adult-like diet at 12 months (11). In 2017, the European Society for Pediatric Gastroenterology, Hepatology, and Nutrition Committee on Nutrition in 2017 recommended that complementary foods should be introduced between 4 and 6 months of age (29). From the neurodevelopmental point of view, lumpy (semisolid) food can be consumed between 6 and 12 months, and after 9 months, most infants can eat finger food and are able to chew their food (29). Northstone et al. reported a tendency toward feeding difficulties and the avoidance of certain foods if solid foods are not introduced until 9–10 months of age (12). Consistent with those studies, we defined the normal expansion of oral diet as the introduction of pureed foods before 9 months of age and of mashed foods and soft lumps before 12 months of age.

Among the 77 infants with FOF at the time of the VFSS in our study, the two raters reported five disagreements between FOIS levels 4 and 5. One such infant was assessed at 270 days of age and was consuming bottled milk. One examiner considered 270 days to be older than 9 months and evaluated the infant at FOIS level 4, whereas the other rater considered the infant younger than 9 months and evaluated him at FOIS level 5. Another infant was mainly consuming bottled milk at 305 days of age and had been attempting a 50-cc volume of pureed food 1 week before the evaluation. In this case, one examiner rated the feeding status as FOIS level 5 because she considered a pureed diet to be normal, whereas the other examiner assigned a rating of FOIS level 4 because the pureed diet had been initiated after 9 months of age. To improve the inter-rater reliability, it could be recommended to give a clear instruction that the FOIS for infants is based on the diet at the time of the evaluation.

## Study Limitations

In this study, we were unable to evaluate the correlation between the FOIS for infants and developmental assessments. Future studies could potentially apply the Bayley Scales of Infant and Toddler Development (30) in conjunction with the FOIS assessment. Additionally, this was a single-center study, which may have led to selection bias. Moreover, the validity and reliability of the FOIS for infants were assessed with a heterogeneous disease group. Crary et al. (7) originally suggested the adult FOIS for stroke patients. Afterwards, other researchers expanded the FOIS for patients with traumatic brain injury (31, 32), head and neck cancer (33, 34), vocal fold immobility (35), vagal schwannoma resection (36), cerebral palsy (37), postsurgical dysphagia (38), neurodegenerative diseases (39), postextubation dysphagia in children (40), and neurogenic dysphagia (41). The FOIS for infants suggested in the present study was a simplified scale with levels reduced from 7 to 5, without taking into account the concepts of single/multiple consistency food, special preparation or compensation, and food restriction. Therefore, the variation in applicability according to disease groups might be less than that in the adult population. However, the scale proposed in this study might be more appropriate for certain disease groups than other groups, and in some groups, this scale would not be applicable. For example, the FOIS suggested in this study could be inappropriate for infants who require continuous total parenteral nutrition because of gastrointestinal problems.

## Conclusions

The FOIS for infants, which reflects the expansion of oral diet in infants, showed adequate reliability and validity. Our findings suggest that this scale could be useful for documenting infants' feeding abilities and evaluating the effectiveness of interventions. The reliability and validity of the FOIS for infants could be improved if caloric contribution as well as the consistency of oral feeding are considered for the distinction between FOIS levels 2 and 3.

## Ethics Statement

All study-related procedures were performed in accordance with the ethical standards of the institutional and/or national research committee and the 1964 Declaration of Helsinki. Ethical approval for the study was obtained from the Seoul National University Hospital Institutional Review Board (IRB) (No. 1807-189-963), which waived the requirement for informed consent due to the retrospective nature of the study.

## Author Contributions

YY: acquisition of data, analysis and interpretation of data, writing and critical revision of manuscript. H-IS: study concept and design, acquisition of data, analysis and interpretation of data, study supervision, and critical revision of manuscript for intellectual content.

### Conflict of Interest Statement

The authors declare that the research was conducted in the absence of any commercial or financial relationships that could be construed as a potential conflict of interest.
